# Optimization and Validation of ELISA for Aflatoxin B1 Detection in Fermented Forages and Feeds

**DOI:** 10.1155/2022/6059880

**Published:** 2022-09-30

**Authors:** Enikő Horváth, Tünde Pusztahelyi, Cintia Adácsi, Ervin Tanyi, István Pócsi

**Affiliations:** ^1^Department of Molecular Biotechnology and Microbiology, Institute of Biotechnology, Faculty of Science and Technology, University of Debrecen, H-4032, Egyetem Tér 1, Debrecen, Hungary; ^2^Central Laboratory of Agricultural and Food Products, Faculty of Agricultural and Food Sciences and Environmental Management, University of Debrecen, H-4032 Böszörményi Street 138 H-4032, Debrecen, Hungary; ^3^Doctoral School of Nutrition and Food Sciences, University of Debrecen, H-4032 Böszörményi Street 138, Debrecen, Hungary; ^4^Romer Labs Diagnostic Ltd, Technopark 5, Tulln 3430, Austria

## Abstract

Enzyme-coupled immunosorbent assays (ELISA) methods are usually validated only for homogenous matrixes like corn and wheat. More complex materials like fermented forages and mixed feed are not targeted for mycotoxin measurement. The low number of ELISA methods found in the literature neither contained the pH set for fermented forages nor dealt with the setting of the matrix:solvent ratio. The sample preparation of these matrixes needs to be optimized and validated for aflatoxin B1 analysis from fermented forages (corn silage and rye haylage) and mixed feed for Romer AgraQuant® Aflatoxin B1 ELISA (Romer Labs, Austria). Drying and pH adjustment of fermented forages had high importance before mycotoxin extraction. Because of the matrix swelling, the 1 : 5 ratio of the sample/extraction solute should have been increased to 1 : 8 to gain the highest aflatoxin B1 recovery. The accuracy and repeatability of the analysis were tested and found to be suitable for further application.

## 1. Introduction

Aflatoxin B1 (AFL B1) measurement possesses worldwide interest as a mycotoxin presents a real threat. Validation of an ELISA method for AFL B1 detection from some food and feed ingredients [1], mainly grain products, are well-known, but the detection of AFL B1 by ELISA from fermented forages and feed mixtures by this rapid test is still a neglected area. However, ELISA applications are good options for the agricultural area as the tests are usually suitable for large numbers of samples and are not costly and time-consuming.

The data suggest that due to the different matrix composition of the feeding stuff, the degree of recovery may greatly vary even for basic materials like grains (e. g., [2] Percent Recovery AgraQuant® Aflatoxin B1 ELISA kit Romer Lab; corn: 91–102%, rice: 70–80%, soybeans: 87–114%, wheat: 87–103%). In the case of fermented forage samples, the presence of lactic acid bacteria may cause additional complications, which besides significantly decreasing the pH, may be able to passively bind aflatoxins in feed materials [3, 4], thereby removing part of the toxin to be detected from the system. Interferences due to the individual matrix of the feed, e. g., low pH, cross-reaction with other aflatoxins, and excessive water absorption of the matrix can occur. The pH of the sample should be 6–8; hence, excessive alkaline or acidic conditions may affect the antigen-antibody binding in ELISA. Therefore, the pH should be adjusted before the test.

We optimized the sample preparation and validated the ELISA method to detect contamination in AFL B1 fermented forages and feed samples using the AgraQuant® Aflatoxin B1 ELISA test kit (Romer Labs, Austria).

## 2. Results and Discussion

### 2.1. Initial pH and a Corresponding Range of Feed Types

The initial pH of the matrixes varied between 4.02 and 6.42, which is determined by the chemical composition of the starting material, such as protein and carbohydrate content, and by the presence of metabolites (e. g., volatile organic acids, lactic acid, and acetic acid) originated from microbes that utilize the nutrient sources. For the ELISA kit to function properly, the pH of the sample must be set to pH 6–8. That may affect subsequent ELISA results, as chemical reactions occur slightly in the matrix during pH adjustment, in which products such as salts and precipitates are formed that may interfere with the ELISA system, so we tried to minimize this effect when adjusting the pH.

We tested two chemicals, one commonly used NaOH and the other NaHCO_3_, which was applied in detecting deoxynivalenol and its conjugates in ELISA (Romer AgraQuant® DON Assay Test Kit; Romer Labs) from beer [5]. Although precipitation was observed with both chemicals, the solution became less cloudy applying 1M NaOH, and a lower volume of the solution had to be added to the system to adjust the correct pH than when 1M NaHCO_3_ solution was used at the same concentration. Therefore, we decided to continue working with 1M NaOH solution.

### 2.2. Extraction Method

The recommended protocol for sample preparation requires the matrix to be minced and then mixed or blended with the extraction agent for three minutes and immediately filtered. However, the blender commonly used for sample preparation is unsuitable for effectively homogenizing fibrous forages. Therefore, the first crucial step of sample preparation was the homogenization of the sample because silage/haylage samples are shredded to different sizes, often containing coarse, hard-to-grind particles. The samples were dried and then shredded to small particle sizes in an industrial grinder. Finally, the dried sample minced to fine particles was used during the spiking.

After the dry sample had been spiked, we decided to carry out the extraction in lockable Falcon tubes (also to prevent evaporation) with a multifunctional shaker for half hour to provide sufficient time and a more effective protocol for recovering the toxin. All dried and spiked matrixes were tested for AFL B1 as quality control and were suitable for further ELISA process.

### 2.3. Sample–Extraction Solute Ratio

With the initially recommended 1 : 5 sample: extraction solution ratio for homogenous and less fibrous matrixes such as corn or wheat and low recovery (below 70%) was detected for the fibrous fermented forages and mixed feed due to significant matrix swelling/liquid uptake. However, we wanted to improve the average recovery to higher than 70%. Furthermore, according to the literature, it is possible to reduce the distortion caused by the matrix effect by diluting the sample [6–9]. Therefore, the dilution in the 1 : 5 ratio determined by the manufacturer for homogenous matrixes with low fibre content was increased up to 1 : 10 (Table 1).

### 2.4. Performance Characteristics of the Analytical Method

#### 2.4.1. Determination of the Limit of Detection and Quantitation

Prepared sample matrixes were tested in the HPLC method to ensure their mycotoxin-free state. From the repeated ELISA measurement of the blank matrix samples, mean concentrations, standard deviations, and limit of detection and quantitation were calculated (Table 2). From the calibration curves gained in ELISA, *r*^2^ values were also checked and found between 0.995 and 0.985.

#### 2.4.2. Accuracy

The mean, standard deviation, and relative standard deviation are calculated by determining the concentration of eight independently measured solutions containing the spiked AFL B1 (Table 3).

#### 2.4.3. Repeatability

Two different operators determined the AFL B1 concentration of the matrixes from the same sample on different days, making three independent measurements per day. The results are used to determine significant differences between the groups by scattering analysis (Tables 4–6). For all matrixes, there were no significant differences within the analysis.

## 3. Discussion

The enzyme-coupled immunosorbent assays (ELISA) methods usually are validated for homogenous matrixes such as corn and wheat, and more complex materials like fermented forages and mixed feed are not targeted because of their complex composition, low pH, and high water content.

The fermented forages need to be dried and ground, and the pH needs to be set before ELISA analysis considering the high water content and the acidic pH of these materials. As the fermented forages and mixed feeds tend to swell in extraction solutions, higher dilution of the samples at a 1 : 8 ratio is recommended to gain a better recovery of the mycotoxin.

Most of the methods we found in the literature neither contained the pH set for fermented forages nor dealt with the setting of the matrix:solvent ratio. The proper ratio is essential to maximise the extraction and avoid the loss of the liquid phase due to matrix swelling, which can be a source of false measurements [10–12]. Maggira et al. [13] set the pH of the extract, but they worked with barley and durum wheat mixture not comparable with a fermented forage. Pirestani et al. [14] dried the silage under the sun, which could cause UV degradation of AFL B1. Makau et al. [15] also validated an AFL B1 ELISA method (RIDASCREEN). However, they did not mention the matrixes they applied in the measurement validation, and they did not study the matrix effect on the assay. Mongkon et al. [16] applied another ELISA system (DOA-Aflatoxin F ELISA Test Kit) and calculated 3.12–6.93 LOQ values, which is higher than for the AgraQuant® Aflatoxin B1 ELISA kit. At the same time, the recovery suitably ranged between 86.6 and 90.7.

Under modified sample preparation, the AgraQuant® Aflatoxin B1 ELISA kit was successfully applied for feed and fermented matrixes with more than 70% recovery. Accuracy and repeatability analyses revealed a suitable process for sample preparation. Relative standard deviation (RSD (%)) was lower than 11% for all tested matrixes. The recovery of AFL B1 was the highest in rye haylage and the lowest in milking cow feed concentrate; however, even for that samples, the recovery of AFL B1 was higher than 70%, which is comparable to that of some grains. Therefore, the method can be suggested as a quality control method.

## 4. Materials and Methods

### 4.1. Romer AgraQuant® Aflatoxin B1 ELISA Test Kit

Romer AgraQuant® Aflatoxin B1 ELISA test kit (Romer Labs, Austria) has a declared quantitative range between 2 and 50 ppb AFL B1 with a 2 ppb limit of detection and a 2 ppb limit of quantitation. The declared reactivity is 100% for AFL B1, 8.4% for aflatoxin B2, 16% for aflatoxin G1, and <0.01% for aflatoxin G2.

This ELISA kit is ideal for measuring multiple samples in parallel, up to 42 samples, with an incubation period of 15 minutes. The kit is manufactured in a ready-to-use format to reduce handling errors. The kit contains five AFL B1 standards (0 ppb, 2 ppb, 5 ppb, 20 ppb, and 50 ppb), antibody-coated plate, conjugate, substrate, and stop solution.

### 4.2. A Detailed Description of the Sample Preparation

Representative samples (at least 1 kg) were gained at the feed producers. Sample preparation was carried out for matrixes with water content higher than 20%, and the matrix was dried at 60°C ± 1°C (LABORMIM LP-402, Hungary) for 48 hours before sample grinding through a 1 mm grid (SM 100, Retsch). 20 g of ground sample was weighed into a clean container (Falcon tube). Contamination of the sample with AFL B1 toxin (Biopure) was carried out to a maximum concentration of 40 ppb.

The mycotoxin was extracted with 70/30 (v/v) (%) methanol/water extraction solution in a 1 : 5 to 1 : 10 ratio shaking with 360° Multi-Functional Tube Rotator (BIO-SAN) for 30 mins (orbital: 60/109 sec, reciprocal: 5°/5 sec, vibro: 5°/1 min). After settling and filtering the extract through Munktell-Ahlstrom 292 filter paper (5-8 *μ*m), the pH of the filtrate was adjusted to pH 6.5 with 1M NaOH or 1M NaHCO_3_ solution.

### 4.3. Detection of Aflatoxin B1 by AgraQuant® Aflatoxin B1 ELISA Test

200 *μ*l of the conjugate solution was loaded into the dilution wells, and then 100 *μ*l of each standard and sample were added to the dilution cavities. Next, the conjugate was mixed with the sample, and 100 *μ*l from the dilution wells was transferred to the antibody-covered wells and incubated at room temperature for 15 minutes. Finally, the wells were washed with 250 *μ*l of deionized water five times (a plate washer can be applied), and the washed wells were dried on a paper towel or aspirated with a plate washer.

100 *μ*l of the substrate solution was added to the plate wells covered with the antibody and incubated at room temperature for 5 minutes (competitive ELISA: blue color is more intensive with lower AFL B1 concentration). The enzyme reaction was stopped with 100 *μ*l stop solution (color change to yellow), reading the absorbance at 450 nm and 630 nm differential filter. We used a Synergy HTX multi-mode reader (BioTek) and Gene5 3.05 software (BioTek) for data collection.

### 4.4. Method of Evaluating the Results

The dose-effect curve was prepared based on the OD values of the standards. Since the amount of AFL B1 is known in each standard, unknowns can be measured by interpolation based on this standard curve if the Log/Logit regression model is used to interpret the results. Calibration curves were prepared for AFL B1 and coefficients of determination (*r*^2^) were calculated in Windows Office 365 Excel software.

### 4.5. Validation

#### 4.5.1. Recovery

Recovery calculation (1):

(1) $$\begin{array}{c} R=\frac{C_{i}}{C_{\text{ref}}}, \end{array}$$

where, *R* is recovery; *C*_i_ is measured value from spiked feed; and *C*_ref_ is measured value from spiked methanol.

#### 4.5.2. Accuracy

For intra-assay precision within one run, an 8-fold determination of the samples was performed. Mean and standard deviation was calculated (2). The relative standard deviation (RSD) as the absolute value of the coefficient of variation (CV) in (%) was also calculated. The aim was to keep the value below 15 RSD (%) (3).

(2) $$\begin{array}{c} s=\sqrt{\frac{1}{N-1}{\left(x_{i}-\bar{x}\right)}^{2}}. \end{array}$$

(3) $$\begin{array}{c} \text{RSD}=\frac{s}{\bar{x}}, \end{array}$$

where, *s* = sample standard deviation; RSD = relative standard deviation; x1,…, *x*_N_ = the sample data set; *x̄* = mean value of the sample data set; and *N* = size of the sample data set.

#### 4.5.3. Repeatability

For the reproducibility (interassay precision) test, AFL B1 was measured by two operators in three repetitions in three different runs from freshly prepared samples. Daily mean and standard deviation were calculated, and the significance tests between the daily results and operators were performed. *t* values were calculated and compared to *t* critical values (*t*_crit_) in the Microsoft Excel program.

Where, |*t*| ≤ *t*_crit_ at *p* ≤ 0.05, the different runs caused an insignificant difference between the measurements.

### 4.6. Mycotoxin Measurement with HPLC-FD Method

All matrix samples and spiked samples were also tested. All HPLC measurements were carried out on Dionex Ultimate 3000 (Thermo Scientific) HPLC equipment. Aflatoxin Mix 1 calibrant solution (Biopure) was purchased as standard from Romer Labs (Austria) and applied in suitable dilutions.

For aflatoxin detection, Phenomenex (Torrance, CA, USA) RP-C18 column (150 *∗* 4.6 mm, 5 *μ*m) was used with Romer UV derivatization unit (Romer Labs, Austria) and a fluorescence detector ex360 nm, em440 nm with methanol: water (45 : 55) eluent.

Performance as the limit of detection (LOD), linear range, and reproducibility of the applied HPLC method was determined with Quality Control Material Aflatoxin B1 in corn Mid-level (Biopure) (*n* = 8). For AFL B1, the LOD was 0.01 ppb, and the linear ranges were up to 300 ppb. In addition, relative standard deviation (RSD (%)) as an absolute value of the variation coefficient was calculated and found under 10% in all cases.

### 4.7. ELISA Limit of Detection and Quantitation

The quality of the assay was assured by the limit of detection (LOD), which was determined experimentally from the concentration of 8 blank matrix samples as the mean concentration of blank samples + 3-fold standard deviation of the concentrations of blank samples. The limit of quantification (LOQ) was determined experimentally from the concentration of 8 blank matrix samples as the mean concentration of blank samples +  9-fold standard deviation of the concentrations of blank samples.

## Figures and Tables

**Table 1:** Sample/extraction buffer ratio affects the aflatoxin B1 (AFL B1) recovery for corn silage. After pH adjustment (pH = 6.5), an average of parallel measurements is shown (CV% < 15%).

Sample: extraction buffer ratio	AFL B1 recovery (%)
1 : 5	68.51
1 : 6	56.66
1 : 7	81.23
1 : 8	114.1
1 : 9	84.59
1 : 10	80.26

**Table 2:** Calculated ELISA data for the limit of the detection and quantification with Romer AgraQuant® aflatoxin B1 ELISA test kit (*n* = 8).

Parameters	Corn silage	Rye haylage	Milking cow feed concentrate
Mean concentration of the blanks (MC) (ppb)	2,146	2,040	2,135
Standard deviation (SD) (ppb)	0,099	0,102	0,098
Limit of detection (LOD) (ppb)	2,443	2,346	2,429
Limit of quantification (LOQ) (ppb)	3,037	2,958	3,017

**Table 3:** Accuracy and recovery calculation of AFL B1 for fermented forages and mixed feed *n* = 8.

	Corn silage		Rye haylage		Milking cow feed concentrate	
	AFL B1 (ppb)	Recovery (%)	AFL B1 (ppb)	Recovery (%)	AFL B1 (ppb)	Recovery (%)
Mean	29.68	90.73	31.66	96.30	32.44	82.14
SD	1.98		3.27		3.51	
RSD (%)	6.67		10.34		10.83	
Evaluation	RSD (%)		RSD (%)		RSD (%)	
	<15%		<15%		<15%	
	Compliant		Compliant		Compliant	

**Table 4:** Repeatability of the ELISA method for corn silage. Two operators measured AFL B1 in three repetitions on three different days from freshly prepared samples. SD, standard deviation. Spike: 35 ppb AFL B1.

Corn silage	Repetition	1st day AFL B1 (ppb)	2nd day AFL B1 (ppb)	3rd day AFL B1 (ppb)
Daily	Mean	29.17	27.04	28.11
	SD	4.30	3.23	1.95

Significance test between days results	*T*	*t *_crit_ (95%)	Result	Evaluation

1-2	0.9669	2.2621	*t* < *t*_crit_	No significant difference
2-3	−0.6907	2.3060	*t* < *t*_crit_	No significant difference
1–3	0.5501	2.3646	*t* < *t*_crit_	No significant difference

Significance test between operators	*t*	*t *_crit_ (95%)	Result	Evaluation

Operator 1-operator 2	−0.1734	2.2281	*t* < *t*_crit_	No significant difference

**Table 5:** Repeatability of the ELISA method for rye haylage. Two operators measured AFL B1 in three repetitions on three different days from freshly prepared samples. SD, standard deviation. Spike: 40 ppb AFL B1.

Rye haylage	Repetition	1st day AFL B1 (ppb)	2nd day AFL B1 (ppb)	3rd day AFL B1 (ppb)
Daily	Mean	37.10	34.58	38.42
	SD	1.95	3.87	1.72

Significance test between days results	*t*	*t *_crit_ (95%)	Result	Evaluation

1-2	1.4222	2.3646	*t* < *t*_crit_	No significant difference
2-3	−1.2458	2.2281	*t* < *t*_crit_	No significant difference
1–3	−2.2205	2.3646	*t* < *t*_crit_	No significant difference

Significance test between operators	*t*	*t *_crit_ (95%)	Result	Evaluation

Operator 1- operator 2	−1.8758	2.1447	*t* < t_crit_	No significant difference

**Table 6:** Repeatability of the ELISA method for milking cow feed concentrate. Two operators measured AFL B1 in three repetitions on three different days from freshly prepared samples. SD, standard deviation. Spike: 40 ppb AFL B1.

Milking cow feed concentrate	Repetition	1st day AFL B1 (ppb)	2nd day AFL B1 (ppb)	3rd day AFL B1 (ppb)
Daily	Mean	32.60	33.77	33.42
	SD	3.91	2.96	1.64

Significance test between days results	*t*	*t *_crit_ (95%)	Result	Evaluation

1-2	−0.5839	2.2621	*t* < *t*_crit_	No significant difference
2-3	0.2544	2.3060	*t* < *t*_crit_	No significant difference
1–3	−0.4723	2.3646	*t* < *t*_crit_	No significant difference

Significance test between operators	*t*	*t *_crit_ (95%)	Result	Evaluation

Operator 1-operator 2	0.9669	2.1447	*t* < *t*_crit_	No significant difference

## Data Availability

The data are available on request.
